# A Novel Approach for Ovine Primary Alveolar Epithelial Type II Cell Isolation and Culture from Fresh and Cryopreserved Tissue Obtained from Premature and Juvenile Animals

**DOI:** 10.1371/journal.pone.0152027

**Published:** 2016-03-21

**Authors:** Mariola M. Marcinkiewicz, Sandy T. Baker, Jichuan Wu, Terrence L. Hubert, Marla R. Wolfson

**Affiliations:** 1 Department of Thoracic Medicine and Surgery, Katz School of Medicine at Temple University, Philadelphia, PA, United States of America; 2 Department of Physiology, Pediatrics and Medicine, Katz School of Medicine at Temple University, Philadelphia, PA, United States of America; 3 Center for Inflammation, Translational and Clinical Lung Research, Katz School of Medicine at Temple University, Philadelphia, PA, United States of America; 4 CENTRe: Collaborative for Environmental and Neonatal Therapeutics, Katz School of Medicine at Temple University, Philadelphia, PA, United States of America; University of Alabama at Birmingham, UNITED STATES

## Abstract

The *in vivo* ovine model provides a clinically relevant platform to study cardiopulmonary mechanisms and treatments of disease; however, a robust ovine primary alveolar epithelial type II (ATII) cell culture model is lacking. The objective of this study was to develop and optimize ovine lung tissue cryopreservation and primary ATII cell culture methodologies for the purposes of dissecting mechanisms at the cellular level to elucidate responses observed *in vivo*. To address this, we established *in vitro* submerged and air-liquid interface cultures of primary ovine ATII cells isolated from fresh or cryopreserved lung tissues obtained from mechanically ventilated sheep (128 days gestation—6 months of age). Presence, abundance, and mRNA expression of surfactant proteins was assessed by immunocytochemistry, Western Blot, and quantitative PCR respectively on the day of isolation, and throughout the 7 day cell culture study period. All biomarkers were significantly greater from cells isolated from fresh than cryopreserved tissue, and those cultured in air-liquid interface as compared to submerged culture conditions at all time points. Surfactant protein expression remained in the air-liquid interface culture system while that of cells cultured in the submerged system dissipated over time. Despite differences in biomarker magnitude between cells isolated from fresh and cryopreserved tissue, cells isolated from cryopreserved tissue remained metabolically active and demonstrated a similar response as cells from fresh tissue through 72 hr period of hyperoxia. These data demonstrate a cell culture methodology using fresh or cryopreserved tissue to support study of ovine primary ATII cell function and responses, to support expanded use of biobanked tissues, and to further understanding of mechanisms that contribute to *in vivo* function of the lung.

## Introduction

The pulmonary alveolar epithelium is composed of two different types of alveolar epithelial cells that cover almost all of the internal surface area of the lung. Alveolar type II cells (ATII) are cuboid in shape and are smaller than alveolar type I cells (ATI). Although there are many more ATII than ATI in the pulmonary alveolar epithelium, they cover a much smaller percentage of the internal surface area of the lung [1]. These cells perform several important roles required for proper function of alveoli. They regulate the metabolism of surfactant, transport ions, and repair alveoli in response to injury. During alveolar repair, ATII cells transdifferentiate into ATI cells [2]. Primary cultures of ATII cells have allowed for further insight into the function(s) of this cell *in vivo* however, there are limitations with existing methodologies.

While previous primary cell culture of ATII cells has been successful in mouse and rat models, the ATII cell phenotype is quickly lost in traditional, submerged cell culture systems. In this regard, ATII cells transform from their cuboid shape to flattened cells, surfactant synthesis and sorting into lamellar bodies is lost [3]. Ideally, *in vitro* models for ATII cell function would be 3-dimensional, include airflow, an air-liquid interface, with tissue stretching and movement to better approximate the *in vivo* environment of these cells. Submerged cell culture does not mimic the *in vivo* environment of these cells and therefore, does not allow for accurate depiction of *in vivo* function of ATII cells [4]. *In vivo*, ATII cells sit in the corner of alveoli between air of the external environment and the capillary endothelial cells. Maintenance of ATII cell phenotype *in vitro* has been markedly improved with the use of air-liquid interface cultures in rat models [5]. The air-liquid interface cultures more closely approximates the *in vivo* conditions and allows for continued expression of surfactant from ATII cells.

The rationale for this study is multi-dimensional. Although marked improvements in primary ATII cell culture have been made in rat and mouse models, primary ovine ATII cell culture from large animals has not progressed. With respect to large animals, the *in vivo* ovine model is widely utilized in understanding mechanisms and treatments of cardiopulmonary diseases across ages [6–10]. Primary ovine ATII cells, in turn, are useful for dissecting mechanisms at the cellular level to elucidate responses observed *in vivo*, and ultimately to better understand human ATII cell morphology and physiology. Previous study of primary ovine ATII cells in early development has been limited to a single time point [11,12]. Refinement of the primary culture method would allow for further experimentation of cell function, response to environmental or pharmacologic exposures (i.e. tobacco smoke, low or high atmospheric pressure, altered oxygen concentration), and age-dependent differences of these responses. A further rationale for our study, is that biobanked cryopreserved tissue would increase availability and expand utility of tissues harvested following *in vivo*. To address these issues, we present a new approach allowing for isolation of viable ovine ATII cells from fresh and cryopreserved tissue, with preserved morphology and ability to produce surfactant proteins under normal and hyperoxic conditions, and evaluate differences between submerged and air-liquid interface systems for up to 7 days in culture.

## Materials and Methods

Elastase, fetal bovine serum (FBS), penicillin/streptomycin solutions, Glutamine, Amphotericin B, and normal goat serum were purchased from Gemini Bio Products. DNaseI, Dexamethasone, 8-Bromoadenosine 3′,5′-cyclic monophosphate sodium salt (8-Br-cAMP), 3-isobutyl-1-methylxanthine (IBMX), dithiothreitol (DDT), and Gentamicin were purchased from Sigma-Aldrich (St. Louis, MO). Hanks Balanced Salt Solution(HBSS) buffer, Dulbecco’s Modified Eagle Medium (DMEM)/F12 medium, and Transwell 12mm inserts with polyester membranes (pore size 0.4μm) were obtained from Corning Inc. (Lowell, MA). Recombinant human keratinocyte grow factor (KGF) was purchased from Peprotech (Rocky Hill, NJ). Matrigel was purchased from BD Biosciences (Franklin Lake, NJ). Rabbit anti–bovine surfactant protein A (SP-A) polyclonal antibody and goat anti-rabbit IgG Horseradish Peroxidase (HRP) conjugated secondary antibody were purchased from Millipore,(Billerica, MA). Rabbit anti-human pro+mature surfactant protein B (SP-B) polyclonal antibody was purchased from Abcam Inc. (Cambridge, MA), rabbit anti mouse pro surfactant protein C (SP-C) polyclonal antibody was from Seven Hills Bioreagents (Cincinnati, OH), Alexa Fluor488 donkey anti-rabbit IgG secondary antibody and Prolong Gold antifade reagent with DAPI (mounting medium) was from Molecular Probe (Eugene, OR). Mouse anti-human GAPDH antibody was purchased from BioRad (Hercules, CA) and sheep anti-mouse IgG HRP conjugated secondary antibody was purchased from GE Healthcare U.K. Limited (Buckinghamshire, UK). mRNA purification RNeasy mini kit was purchased from Qiagen Inc. (Valencia CA), Maxima SYBR Green qPCR Master Mix and RevertAid first strand cDNA synthesis kit from Thermo Scientific (Waltham, MA) and BCA Protein Assay Kit and SuperSignal West Pico chemiluminescent substrate were purchased from Pierce, (Rockford, IL).

### Isolation and Culture of Ovine Alveolar Type II Cells

#### Tissue origin

Lung tissue was isolated from sheep (n = 14; premature (n = 7): 128 days gestation; juvenile (n = 7): 6 months) after intubation and 4 hrs of mechanical ventilation with supplemental oxygen. Prematurely delivered sheep at 128 days gestation are considered surfactant deficient and were treated with exogeneous surfactant replacement therapy ((lucinactant, Discovery Laboratories, Inc., Warrington, PA) within 10 mins following delivery. Sheep were humanely euthanized (sodium pentobarbitol; 100 mg/kg) according to the Institutional Animal Care and Use Committee (IACUC) at Temple University School of Medicine and performed in accordance with National Institutes of Health guidelines. The same IACUC approved the entire study.

Lung excision was performed using sterile scissors and forceps. Excised lung tissue was placed in sterile tubes containing sterile saline solution. After the large airways were carefully removed, the tissue was cut into 2–3 cm^2^ pieces, washed 3x to remove blood, and immediately processed or cryopreserved using the protocols below.

#### Cryopreservation of lung tissue

Tissue was washed in sterile HBSS/pen/strep/amphB buffer and cut into 2–3 cm^2^ cubes using a sterile scissors and sterile forceps. Cubes were washed again in HBSS/penicillin/streptomycin/amphotericin B and transferred into a sterile 30 ml Luer lock syringe containing 10–15 ml freshly prepared sterile cryopreserving medium (CM):DMEM/F12 supplemented with 100 U/ml penicillin, 100 U/ml streptomycin, 2.5μg/ml amphotericin B, 20% FBS, 10% high quality dimethyl sulfoxide (DMSO). After removing all air from the CM/cube mix, the syringe chamber was closed with a stopcock. Partial vacuum/tissue expansion and perfusion was performed as previously described [13]. Briefly, partial vacuum was created in the syringe chamber by slowly pulling the plunger outwards to increase chamber volume and inwards to return to atmospheric pressure thus to the original chamber volume. Visible gas, released from tissue airways after the syringe was returned to its original pressure, was removed from the syringe chamber by opening the stopcock. This cycle was repeated a total of 5-7x to insure complete perfusion. Perfused tissue pieces were transferred into cryovials and slowly frozen in a styrofoam container in a -80°C freezer. After 24 h, all vials were moved to the liquid nitrogen tank for long term storage. After 2–8 weeks, the tissue was rapidly thawed in a 37°C water bath and placed in HBSS/pen/strep/amphB. Once thawed, the tissue was prepared using the cell isolation protocol described below.

#### Cells isolation and maintenance of alveolar type II cells in culture

Tissue pieces were transferred into individual sterile 30 ml syringes and perfused with sterile HBSS/pen/step/amphB buffer to remove any residual air and blood from the small airways. Perfused tissue was cut into 1 mm pieces using a McIlwain tissue chopper, washed in a sterile beaker with HBSS/penicillin/streptomycin/amphotericin B buffer (HBSS/pen/strep/amphB buffer) containing 10mM DTT to remove mucus, and then strained through 3 layers of sterile gauze. The wash-strain cycle was repeated 3x with HBSS/pen/strep/amphB buffer containing 10 mM DTT and then 3x with this buffer without DTT. Strained tissue was digested in DMEM-F12 elastase (2 mg/ml) solution with gentle rocking for 45min in an incubator at 37°C and air/5% CO_2_. Digestion was stopped by adding an inhibition solution: 10% FBS and 120U/ml DNaseI in DMEM-F12 medium. The digested mixture was triturated for approximately 5 min with a 25 ml pipette to break up clumps, filtered through sterile gauze, and then through a 40 μm cell strainer. The crude alveolar cell suspension obtained after the last filtration was diluted 1:1 in HBSS/pen/strep/amphB buffer containing 10 mM DTT and centrifuged at 200g for 10 min. Cell pellets were washed 2x with HBSS/pen/strep/amphB buffer without DTT. Four repeated steps of adherence were performed. First, cells were resuspended in DMEM-F12 containing penicillin/streptomycin/amphotericin B, but without FBS, transferred to Petri dishes coated with sheep IgG and incubated for 60 min at 37°C in air/5% CO_2_ in order to allow nontype II cells to attach [5,14]. Then, nonadherent type II cells were gently collected, resuspended in DMEM-F12 containing penicillin/streptomycin/amphotericin B and 10% FBS, plated in culture dishes and incubated for another 45 min. This cycle was repeated 3x.

After the last incubation, nonadherent ATII cells were gently collected, centrifuged and resuspended in a 1:1 (v/v) mixture of NH_4_Cl lyses buffer and HBSS/pen/strep/amphB buffer to lyse red blood cells, and then washed 2x with HBSS buffer. Obtained ATII cells were resuspended in growth medium containing: 10% FBS, 100 U/ml penicillin, 100 U/ml streptomycin, 2.5 μg/ml amphotericin B, 10 ng/ml gentamicin, supplemented with KGF 10 ng/ml and DCI additive (10nM dexametahasone, 0.1mM 8-Br-cAMP, 0.1mM IBMX) to promote cell attachment, proliferation and differentiation and seeded in Transwell inserts coated with 1:10 Matrigel to allow them to attach to the permeable membrane.

After 48h, (beginning of d3 of culture) when ~90% of cells were attached to the membranes, medium and unattached cells were removed; adhesion medium in the apical and bottom of each insert was exchanged for serum-reduced medium containing 2% FBS and all supplements described above. Then, in half of the inserts, medium was removed from the apical part to create an air-liquid interface culture. Cells were maintained for the next 4 days in both submerged and air-liquid interface wells. Medium was changed every 48h.

In this way, freshly isolated ATII cells were cultured in parallel as a submerged system (S) and as an air-liquid interface system (A). In the S system, from the day of isolation (d0), cells were maintained for 7 days on a permeable membrane with medium in the apical and bottom part of the inserts. The air-liquid interface culture was created on d3 of submerged culture by removing the medium from the apical face of the inserts, creating A0. Cells were maintained in those conditions for the next 4 days. Cells were labelled by total days of air-liquid interface culture (A0-A4) and submerged culture counterpart (S).

### Hyperoxia

To create a hyperoxic environment, a sterile resealable, compliant plastic bag was placed in a cell culture incubator. Tubing connectors were integrated into the 2 sealed corners of the bag to maintain a tight seal. Tubing from the gas supply (95% O_2_ /5% CO_2_) was connected to the inlet of the bag and tubing from the outlet of the bag was placed at the surface of a water-filled beaker. This system allowed visualization of continuous flow without generating positive pressure within the compliant bag. On the indicated day of air-liquid interface culture, Transwells with ATII cells isolated from fresh or cyropreserved ovine lung tissue, were placed in the sealed, sterile plastic bag and exposed to the gas supply as described above. After 24h and 72 h exposure, the cells were removed from the bag, washed 3x with PBS, lysed with RIPA or RLT buffer and analyzed by Western blot or qPCR, respectively.

### Assessment of Cell Viability and Purity

Viability of freshly isolated cells was determined by Trypan blue exclusion [15]. Purity of isolated ATII cells was determined by the modified Papanicolaou dark-blue-purple stain classically used for ATII identification through detection of lamellar bodies according to the protocol as described by Dobbs [14,16]

### Western Blot Analysis

On the indicated day of culture, growth medium was removed from the inserts; cells were washed 3x with sterile PBS and lysed in inserts using sterile RIPA buffer. Total protein concentration in cell lysate was determined with the Pierce BCA Protein Assay Kit. Samples, with matched protein concentration, were separated accordingly on 12% polyacrylamide gel for 1h at 200V. Proteins were transferred onto nitrocellulose membranes at 94V for 2h. Membranes were blocked and incubated with anti SP-B primary antibody and anti-GAPDH primary antibody, followed by secondary HRP conjugated antibody in the Snap i.d. system (EMD Millipore; Darmstadt, Germany). The protein bands were visualized with chemiluminescent substrate and camera, and quantitated using densitometry to analyze isolated ATII cell SP-B and GAPDH protein expression in submerged and air-liquid interface culture systems over time as a percentage of that on the day of cell isolation (d0).

### Immunocytochemistry

On the indicated day of culture, growth medium was removed from the inserts, cells were washed 3x with PBS and fixed with 4% paraformaldehyde in 0.1M phosphate buffer for 20 min. After washing 3x with PBS, cells were made permeable by incubation with 0.2% Triton x-100 plus 1% normal goat serum in PBS, on ice for 5 minutes. With the cells still in the wells, they were blocked for 1h with 10% normal goat serum in 1% BSA-PBS, cells, and then incubated for 2h at room temperature with either primary anti SP-A or anti SP-C antibody, each diluted to 1:200 with 1%BSA-PBS. Then the cells were washed 3x with PBS and incubated for 1h with the secondary FITC conjugated antibody at 1:500 dilution with 1% BSA-PBS. After the final wash, the membrane was cut out of the insert, placed on a slide and mounted with DAPI-containing mounting medium. The antibody distribution was visualized with a fluorescence microscope.

### Quantitative PCR

On the indicated day of culture, growth medium was removed from the inserts and cells were washed 3x with PBS and lysed with RLT buffer from mRNA extraction kit (Qiagen). Total mRNA was isolated according to kit instructions and reverse transcribed using RevertAid First Strand cDNA Synthesis Kit (Thermo Scientific). The cDNA obtained was mixed with SYBR Green PCR Master Mix (Thermo Scientific) and surfactant protein gene specific primers for qPCR. 18s expression served as an internal control. The levels of mRNA expression were determined using Expender Relapse Master Cycler quantitative PCR system (Eppendorf). On each plate, standard curves were performed for each surfactant protein and 18S, using a sample to be evaluated on that specific plate. Absolute quantification methods were used from PCR signals; data is presented as the magnitude of mRNA expression of each target gene relative to the expression of 18s, the internal control [17–19].

### Statistical Analysis

Data were analyzed by multi-factorial analysis of variance followed by Bonferroni multiple comparison post-hoc testing to evaluate significance between tissue of origin, culture system, and days in culture. Data are expressed as means ± SEM; significance was accepted at p < 0.05.

## Results

### Viability, Purity, Identification and Yield of Primary Ovine Alveolar Type II Cells in Culture

**Fig 1:**
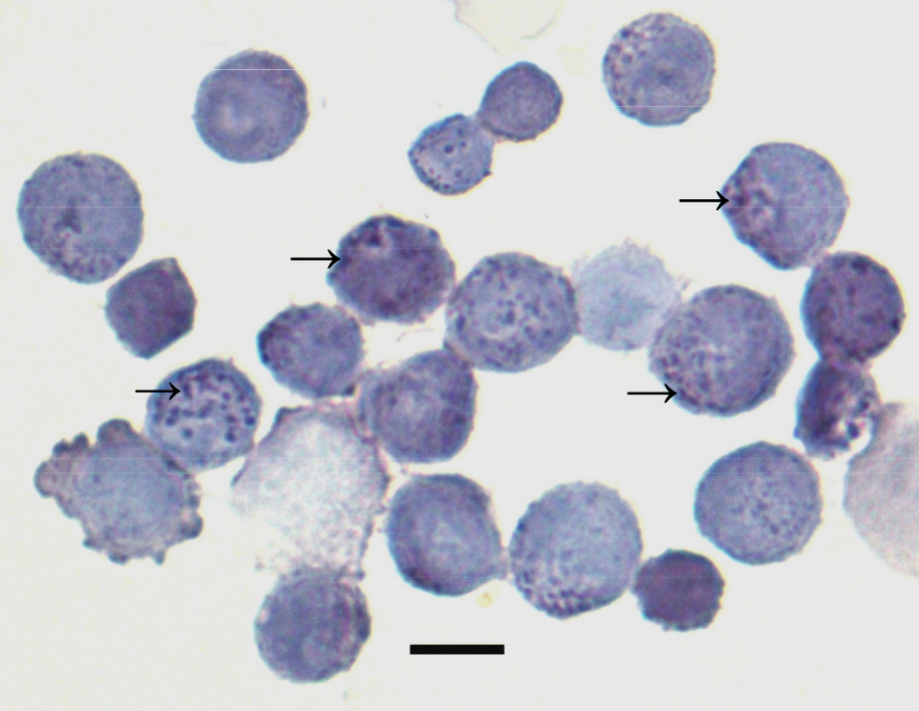
ATII cell identification. Freshly isolated cells were stained according to modified Papanicolau protocol. Type II cells are identified by the presence of dark purple inclusions (arrows) representing lamellar bodies. Purity of ATII cell preparations as assessed using this method was comparable for fresh (85.4 ± 2.2% SD; n = 10 isolations; shown in figure) as compared to cryopreserved tissue (85.1 ± 1% SD; n = 10 isolations); bar = 10μm.

Viability of isolated cells from fresh (90 ± 0.50% SEM; n = 10 isolations) and from cryopreserved (88 ± 1% SEM; n = 10 isolations) tissue was comparable as assessed by Trypan blue exclusion. The morphological hallmark of ATII cells, the surfactant containing lamellar body, was seen by light microscopy as dark blue-purple inclusions using the modified Papanicolau protocol (Fig 1). Using this approach, we assessed the purity of our culture system for ATII cells isolated from fresh (85.4 ± 2.2% SEM; n = 10 isolations) and cryopreserved (85.1 ± 1% SEM; n = 10 isolations) to be comparable. Yield of ATII cells per gram of chopped lung tissue for fresh tissue was 1.9 x 10^6^ ± 0.17 SEM (n = 7 isolations) and for cryopreserved tissue was 1.6 x 10^6^ ± 0.30 SEM (n = 7 isolations). These data indicate that cell viability and yield were not related to the origin of the tissue (ie. fresh vs cryopreserved tissue).

### Surfactant Protein Production of Primary Ovine Alveolar Type II Cells

To further confirm that these cells were ATII, we took a two-prong approach to assess the phenotype to produce surfactant proteins. We focused on SP-B using Western Blotting since this protein is a hallmark of ATII cells [20], and on SP-A and SP-C using immunocytochemistry since these proteins are known to present in specific patterns within the ATII cells [21–24].

**Fig 2:**
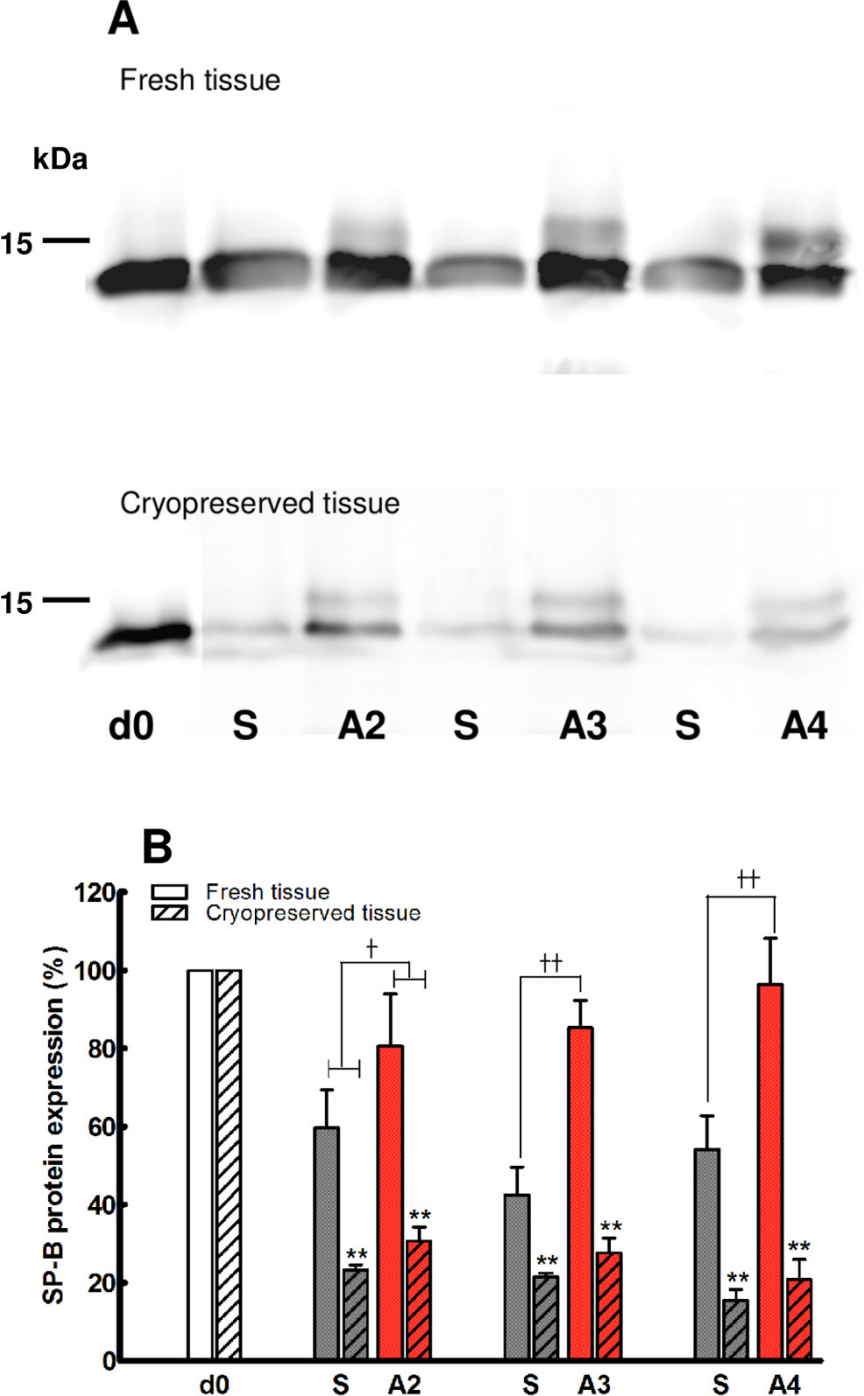
Surfactant protein B (SP-B) expression of ATII cells in culture. A) SP-B protein production was analyzed by Western Blot; B) SP-B protein bands were quantitated by densitometry and expressed as a % of that produced on the day of isolation (d0). Open bars: fresh tissue; hatched bars: cryopreserved tissue; S: submerged culture; A: air-liquid interface culture. ** p < 0.01 fresh vs cryopreserved; Ṫ p < 0.05; ṪṪ p < 0.01 submerged vs air-liquid interface culture systems; mean ± SEM; n = 4/group.

**Fig 3:**
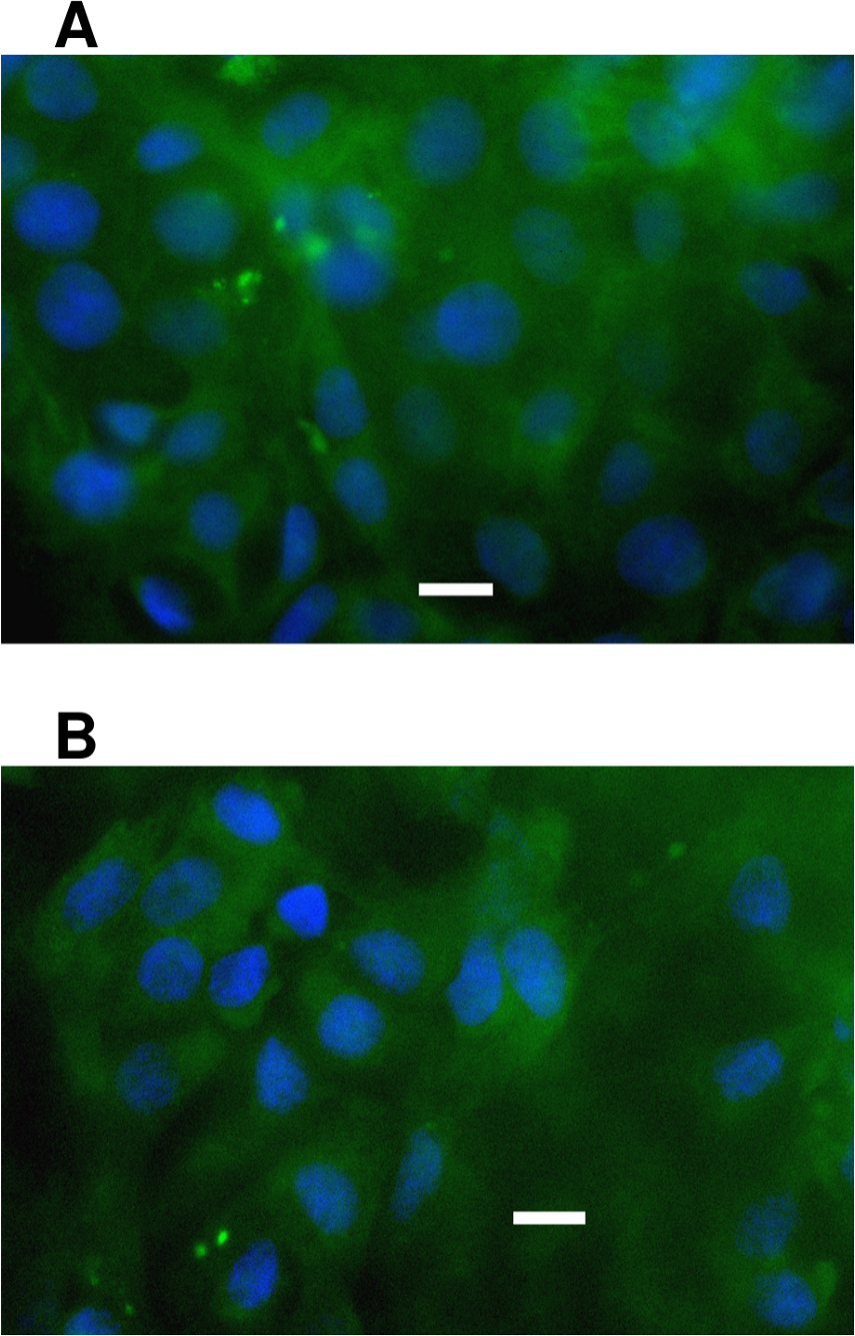
**Visualization of (A) Surfactant protein A (SP-A) and (B) SP-C expression by ATII cells cultured for 6 days.** Cells isolated from cryopreserved tissue and cultured in the air-liquid interface system, were fixed and labeled with primary anti SP-A or SP-C antibody and secondary FITC conjugated antibody, and visualized by fluorescence microscopy; bars = 10μm.

As shown in Fig 2A, ATII cells isolated from fresh or cryopreserved ovine lung tissue, and then maintained in either submerged (S) or air-liquid interface (A) culture systems, demonstrated SP-B production through 7 days post isolation. As quantitated in Fig 2B, cells isolated from fresh tissue showed greater (p < 0.001) and more sustained production of SP-B than those from cryopreserved tissue, independent of the cell culture system. In this regard, as shown by the column pair "S and A4" on the blots, SP-B production remained robust in cells from fresh tissue through day 7 post isolation but markedly diminished by day 6 post isolation in cells obtained from cryopreserved tissue In addition, SP-B production of cells cultured in the air-liquid interface was significantly greater (p < 0.01) than that of submerged cell culture, independent of whether the cells were isolated from fresh (p < 0.001) or cryopreserved (p < 0.05) tissue. It is noteworthy that SP-B protein expression in the cells isolated from fresh tissue and cultured in the air-liquid interface system remained within range of that of the day of isolation, while those cultured in submerged systems remained significantly lower than that of the day of isolation. While the SP-B of cells from cryopreserved tissue was greatly diminished relative to that of cells derived from fresh tissue, immunocytochemistry evidence of SP-A (Fig 3A) and SP-C (Fig 3B) provides additional support that the ATII cell phenotype of these cells remained through 6 days post isolation.

### Hyperoxia

**Fig 4:**
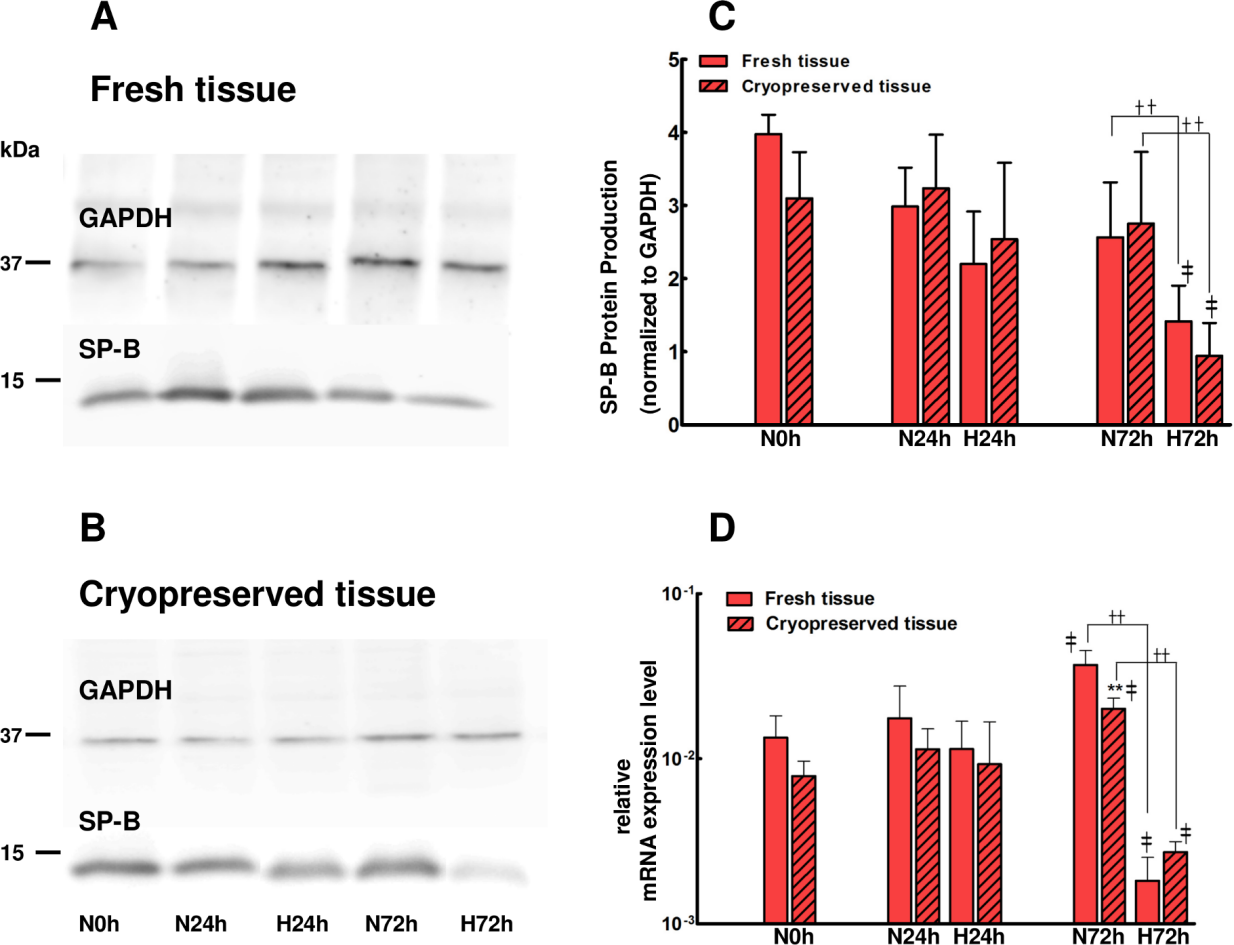
Comparison of the production of SP-B during hyperoxia vs normoxia exposure of air-liquid culture system of ATII cells. Derived from (A) fresh and (B) cryopreserved tissue show time-dependent differences in the amount of SP-B protein as shown by Western Blot and (C) when normalized to GAPDH by densitometry and in (D) SP-B mRNA expression (absolute quantification of SP-B gene relative to 18S as described in methods). N: normoxia; H: hyperoxia; open bars: fresh tissue; hatched bars: cryopreserved tissue; mean ± SEM; n = 4 / group. ** p < 0.01 fresh vs cryopreserved; ṪṪ p < 0.01 normoxic vs hyperoxic 72 h; ‡ p < 0.01 vs 24 h of matched tissue origin (fresh, cryopreserved) and oxygen (normoxia, hyperoxia) condition. bar = 10μm.

ATII cells isolated from fresh or cryopreserved juvenile lung tissue and cultured in air-liquid interface conditions, were exposed to 24h and 72h of hyperoxia and analyzed by Western blotting for SP-B protein expression and by qPCR for SP-B mRNA expression. In order to assess the effect of hyperoxia beginning with sustained levels of SP-B production, cells isolated from fresh and cryopreserved tissues were assessed on the 4th and 3rd day of air-liquid interface, respectively. As shown in Fig 4, ATII cells isolated from either fresh or cryopreserved tissues, demonstrated a time-dependent difference in response to hyperoxia. As compared to normoxic conditions, SP-B protein production(Fig 4A–4C) and SP-B mRNA expression (Fig 4D) trended to be lower by 24 h of hyperoxia; this effect was slightly greater for cells isolated from fresh as compared to cryopreserved tissue. By 72h normoxia exposure, SP-B protein production trednded to decrease, whereas SP-B mRNA expression increased (p < 0.01) in cells isolated from either fresh or cryopreserved tissue. In contrast, by 72h exposure to hyperoxia, SP-B protein production and SP-B mRNA expression decreased markedly, both compared to 24h hyperoxia (p < 0.01) and compared to normoxia 72h controls (p < 0.01).

### Surfactant Protein Gene Expression of Primary Ovine Alveolar Type II Cells

**Fig 5:**
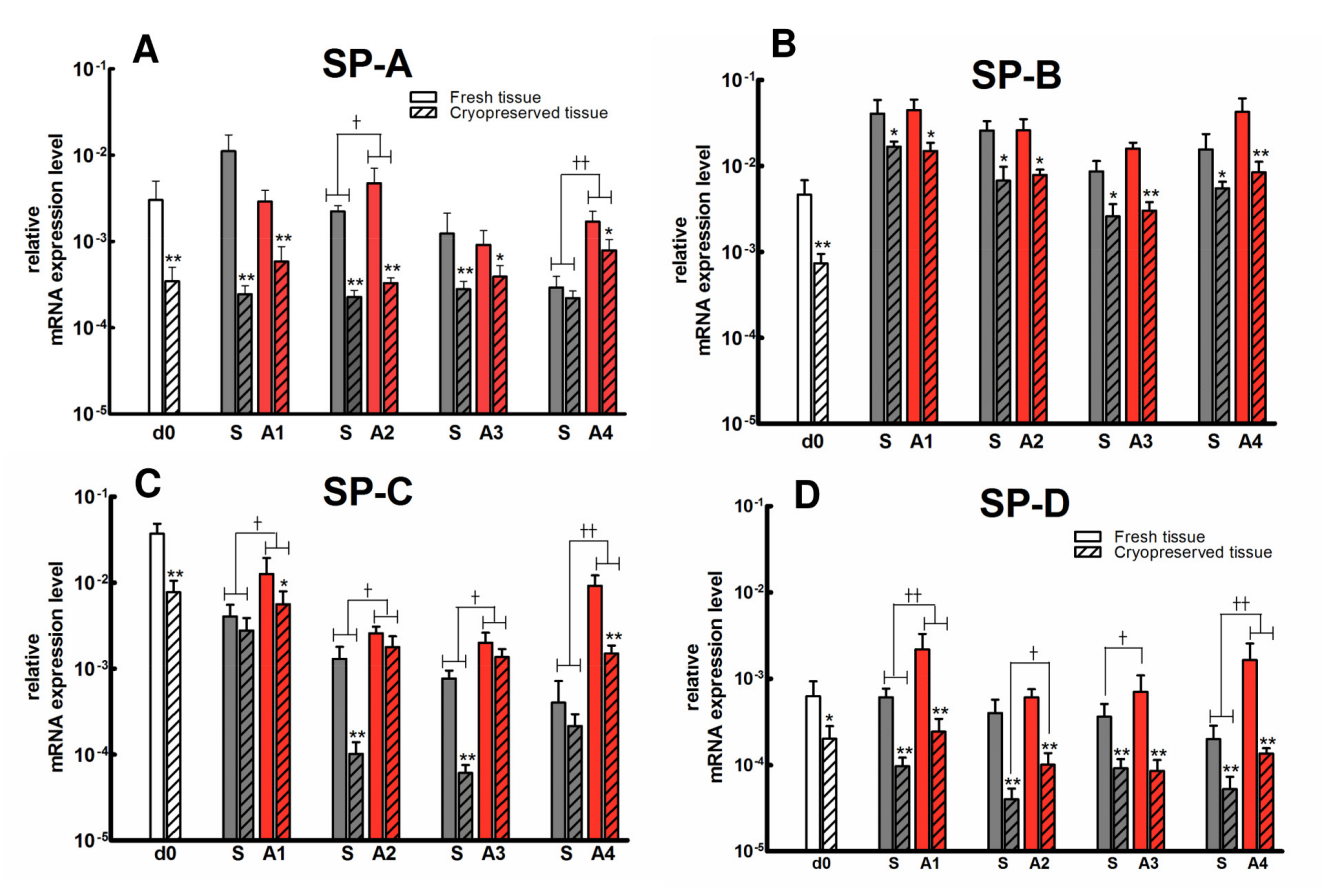
**Surfactant proteins A, B, C, and D mRNA expression during ATII cell culture (absolute quantification of SP gene relative to 18S as described in methods).** d0: day of isolation; S: submerged culture; A: air-liquid interface culture; open bars: fresh tissue (n = 6/group); hatched bars: cryopreserved tissue (n = 8/group); mean ± SEM; * p< 0.05 and ** p < 0.01: fresh vs cryopreserved; Ṫ p < 0.05 and Ṫ Ṫ p < 0.01: submerged vs air-liquid interface culture systems.

Gene expression of surfactant proteins A, B, C, and D of cells isolated from fresh and cryopreserved tissue and cultured in submerged or air-liquid is shown in Fig 5. Across all SPs, gene expression was significantly (ANOVA; p < 0.001) greater in cells isolated from fresh as compared to cryopreserved tissue, independent of the method of culture and day of study. As compared to the submerged cell culture system, cells cultured in the air-liquid interface system demonstrated significantly greater SP-C (Fig 5C; ANOVA; p = 0.01), independent of the day of study and origin of the tissue (i.e. fresh vs cryopreserved) and SP-D gene expression, independent of the day of study but dependent upon origin of the tissue (Fig 5D; ANOVA; p = 0.02). For SP-A gene expression, a similar pattern for culture systems was observed but was dependent upon the day of study.

Relative to the day of isolation, there was a time-dependent difference in SP gene expression that was dependent upon the specific gene, method of culture, and the tissue of origin from which the cells were isolated. In this regard, for SPs A (Fig 5A) and C (Fig 5C) measured in cells isolated from fresh tissue and cultured in the submerged system, gene expression decreased significantly (ANOVA; SP A; p = 0.02; SP C; p< 0.001) over time. Cells isolated from cryopreserved tissues and cultured in the submerged systems as well as cells isolated from fresh or cryopreserved tissue and cultured in the air-liquid interface system showed little difference in SP-A or C gene expression over time. For SP-B (Fig 5B), gene expression in cells isolated from either fresh or cryopreserved tissue and then cultured in either submerged or air-liquid interface increased significantly (p < 0.001) compared to the day of isolation, remained greater through the 7th day post isolation, with little change while in culture. A similar profile was noted for SP-D with the following exceptions. Relative to the day of isolation, SP-D gene expression decreased (p < 0.05) overtime for cells isolated from fresh tissue and cultured in the submerged system, was lower (p < 0.05) but remained constant in cells isolated from cryopreserved tissue and then cultured in the submerged system, higher (p < 0.01) but relatively constant over time for cells isolated from fresh tissue and cultured in the air-liquid interface, and relatively constant for cells isolated from cryopreserved tissue and cultured in the air-liquid interface system.

## Discussion

Culturing primary alveolar epithelial cells has historically presented challenges. Methodological gaps exist that impact the ability to generate and sustain these cells in a system that most closely represents the *in vivo* environment. In this study, we present a new approach allowing for isolation of viable ovine alveolar type II epithelial cells (ATII) cells from cryopreserved and fresh tissue. The methods yielded preserved ATII cell phenotype with respect to morphology, ability to produce surfactant proteins under normal and hyperoxic conditions, and supported evaluation of differences between submerged and air-liquid interface primary ATII cell culture systems for up to 7 days post isolation.

Cultures maintained on plastic dishes and in medium, rapidly lose markers associated with the ATII cell phenotype *in vivo*. Specifically, differentiated ATII II cells characteristically lose properties related to the surfactant system when the cells are isolated and cultured in monolayer[25]. Synthesis and secretion of surfactant proteins (SPs) decrease with time in culture[26], the ability to sort transfected SPs to lamellar body is lost, and the number of lamellar bodies declines[3,27], followed by morphological changes from cuboidal epithelium seen *in vivo* to a flattened cell shape *in vitro*. Numerous studies have addressed this issue by adding various supplements to growth medium or by plating cells on various substrata. Many of these approaches have been at least partially successful in preserving or restoring surfactant-related properties. It has been shown that addition of dexamethasone, 8-bromo-cAMP, and isobutylmethylxanthine (DCI) to growth medium supports ATII cell differentiation, proliferation and SPs expression [28,29]. Keratinocyte grow factor has been identified as a strong supporter of maintaining ATII cell phenotype by increasing SPs mRNA levels and secretion [30–32]. Culturing ATII cells on matrigel coated membranes [33–35] and under air-liquid interface conditions in which medium is added only to the basolateral surfaces of cells while the apical surfaces are exposed to air[5,36–38], have proven to promote their native cuboidal shape and surfactant production. All of these methods have been previously applied successfully to culture of ATII cells retrieved from fresh rodent or human lung tissue.

In this study, we demonstrated procedures for cryopreservation of lung tissue from preterm and juvenile sheep, isolation of viable ATII cells from both fresh and cryopreserved tissue, and subsequent culturing of these cells in both submerged and air-liquid interface systems providing cell models to assess function over multiple time points. Cell viability and ATII cell purity following isolation from both fresh and cryopreserved tissue was consistent with previous studies [11,12]. Key components of our approach was that growth medium was supplemented with DCI, KGF and 2% FBS, cells were seeded in Transwells on permeable membranes previously coat with 1:10 diluted matigel and maintained in submerged or air-liquid interface conditions. These optimized culture conditions resulted in ongoing expression of surfactant proteins (SP-B protein and gene expression and SP A, C, and D gene expression) up to 7 days in cells derived from fresh and cryopreserved lung tissue as well.

SP-B is a distinctive ATII cell marker (24, 25). *In vivo*, SP-B is normally secreted by ATII cells, is required to maintain alveolar integrity to avoid lung collapse, and its absence and insufficiency are associated with increased risk of death and morbidity, respectively [24,39–41]. The successful isolation and culture of ATII cells expressing SP-B from ovine preterm and juvenile, fresh or cryopreserved lung tissue confers a methodological advancement for the ability to utilize ATII cell studies *in vitro*. In cells retrieved from fresh lung tissue, SP-B production was sustained up to 7 days post isolation. The highest level of SP-B production of cells in culture, comparable to that in freshly isolated cells, was seen on the 4th day of air-liquid interface system; whereas, cells derived from the same fresh lung tissue but cultured in submerged system showed consistently declining levels of surfactant protein SP-B expression over time. In comparison to cells from fresh tissue, cells retrieved from cryopreserved lung tissue, although producing less SP-B than those from fresh tissue, demonstrated sustained SP-B production for 6 days post isolation. While not reaching levels comparable to that of freshly isolated cells, the highest levels of SP-B production of cells from cryopreserved cells was seen on the 3rd day of air liquid interface. Similar to cells derived from fresh tissue, cells derived from the same cryopreserved lung tissue cultured in submerged system showed consistently declining levels of SP-B production over time. These findings indicate that primary ovine ATII cells obtained from either fresh or cryopreserved tissue are capable of producing SP-B whether cultured in submerged or air-liquid interface. The sustained levels of SP-B production of ATII cells in the air-liquid interface culture (7 days post isolation for cells from fresh lung tissue; 6 days post isolation for cells from cryopreserved lung tissue) indicate ongoing synthesis of SP-B demonstrating preserved ATII cell phenotype. These findings are relevant as literature evidence of *in vitro* secretion of SP-B by cultured primary cells is limited at best, and requires at least 5–7 days of culturing prior to evidence of SP-B production.

Despite little difference in cell yield between fresh and cryopreserved tissue, both protein and mRNA of SP B, and mRNA of SP A, C, and D was decreased from cells derived from cryopreserved as compared to fresh tissue. It is noteworthy that similar effects on protein production and gene expression in cells obtained from other tissues have been reported [42]. The reason(s) for this is unclear. While mechanistic assessment of this observation is beyond the scope of the present study, possible explanations may be related to the rates of cooling and thawing. We attempted to minimize this effect by controlling the freezing rate with DMSO and thawing rate by a controlled temperature environment. From studies of freezing cells that are in suspension, it is known that because water crystallizes in the extracellular space, a solute concentration gradient is created across the membrane. Slow cooling exacerbates this effect, water moves out of the cells, the cells dehydrate, shrink and the membranes are damaged. If cooling is too fast, intracellular ice forms, again causing membrane damage. Other possible explanations may be related to freeze-thaw induced oxidative stress and production of reactive oxygen species (ROS), imbalance between ROS and antioxidants, molecular damage to DNA, proteins and lipids of the cells derived from these tissues.

The air-liquid interface culture system of ATII cells more closely approximates the *in vivo* conditions as compared to the submerged system. We found that the impact of culture systems on protein and gene expression of primary ovine ATII cells was similar whether the cells were derived from fresh or cryopreserved tissues. Specifically, we observed greater surfactant protein, and greater and more sustained surfactant protein gene expression in the air-liquid interface as compared to the submerged system. This finding is consistent with previous studies of ATII cells isolated from fresh rodent lung [5]. Importantly, this finding is consistent with changes in the *in vivo* surfactant profile during transition from the fluid to air-filled lung [43].

Our findings also provide important guidance for the experimental use of cultured primary ovine ATII cells. Relative to the day of isolation, we found a time-dependent difference in SP gene expression that was dependent upon the specific gene, method of culture, and the tissue of origin from which cells were isolated. As an example, the magnitude and time-dependent difference in the SP-B protein production across each of these systems should be factored into experimental expectations when assessing the impact of stimuli on SP-B production. More specifically, it is important to consider the conditions and cell culture day where production is comparable under the control, non-stimulated setting. In this regard, in order to assess the effect of hyperoxia beginning at comparable time-points of sustained SP-B production, we assessed cells isolated from fresh and cryopreserved tissues on the 4th and 3rd day of air-liquid interface, respectively. Using this approach, we were able to show that the effect of hyperoxia on cells derived from cryopreserved tissues and then cultured in the air-liquid interface system to be relatively the same as that on cells derived from fresh tissue and cultured in the same way.

Biobanks of frozen or fixed ovine lung tissues obtained following *in vivo* protocols have, historically, proven useful to assess molecular/biochemical and structural biomarkers, from tissue homogenates or sections, respectfully. However, use of these banked tissues for primary alveolar epithelial cell culture for dissection of mechanisms or treatment effects on the cellular level has been limited. We have demonstrated practical and productive methods of tissue cryopreservation, cell isolation and culturing with subsequent protein and gene expression of surfactant proteins reflecting metabolically active ATII cells. We anticipate that these methods will prove useful to support more prolonged study of primary ovine ATII cells and will be translatable to support human primary ATII cell cultures.
